# Elimination of glutamatergic transmission from Hb9 interneurons does not impact treadmill locomotion

**DOI:** 10.1038/s41598-021-95143-y

**Published:** 2021-08-06

**Authors:** Lina M. Koronfel, Kevin C. Kanning, Angelita Alcos, Christopher E. Henderson, Robert M. Brownstone

**Affiliations:** 1 Department of Medical Neuroscience, Faculty of Medicine, Dalhousie University, Halifax, NS B3H 4R2 Canada; 2 Center for Motor Neuron Biology and Disease, Columbia Stem Cell Initiative, Columbia Translational Neuroscience Initiative, Columbia University, New York, NY 10032 USA; 3 Department of Pathology and Cell Biology, Neurology, and Neuroscience, College of Physicians and Surgeons, Columbia University, New York, NY 10032 USA; 4 Department of Neuromuscular Diseases, UCL Queen Square Institute of Neurology, UCL, London, WC1N 3BG UK; 5 Present Address: Okinawa Institute of Science and Technology Graduate University, 1919-1 Tancha, Onna-son, Kunigami-gun, Okinawa, 904-0495 Japan; 6 Present Address: Regeneron Pharmaceuticals, 777 Old Saw Mill River Rd, Tarrytown, NY 10591 USA; 7 Present Address: Biogen, Inc., 225 Binney St, Cambridge, MA USA

**Keywords:** Neuroscience, Physiology

## Abstract

The spinal cord contains neural circuits that can produce the rhythm and pattern of locomotor activity. It has previously been postulated that a population of glutamatergic neurons, termed Hb9 interneurons, contributes to locomotor rhythmogenesis. These neurons were identified by their expression of the homeobox gene, Hb9, which is also expressed in motor neurons. We developed a mouse line in which Cre recombinase activity is inducible in neurons expressing Hb9. We then used this line to eliminate vesicular glutamate transporter 2 from Hb9 interneurons, and found that there were no deficits in treadmill locomotion. We conclude that glutamatergic neurotransmission by Hb9 interneurons is not required for locomotor behaviour. The role of these neurons in neural circuits remains elusive.

## Introduction

Over the past decade, genetic knowledge has been increasingly harnessed to dissect neural circuits that produce behaviour^1^. This knowledge, for the most part, has been derived from our understanding of neural development^2^. In the spinal cord, expression patterns of transcription factors have been discovered and cardinal classes of spinal interneurons defined, enabling the development of tools that have been used first to identify and then to functionally alter neuronal populations. These tools have led to concepts of spinal motor circuit organisation and the roles of specific classes of interneurons in producing motor output^3^.

One critical homeobox gene expressed during development is Hb9 (Mnx1—motor neuron and pancreas homeobox 1). Hb9 is crucial for consolidation of spinal motor neuron (MN) fate during development, and is expressed in somatic MNs as well as spinal visceral MNs (sympathetic preganglionic neurons, SPNs)^4^. Thus a transgenic mouse in which expression of enhanced green fluorescent protein (eGFP) was driven by the promoter for Hb9 (Hb9::eGFP) was made for the selective study of motor neurons^5^. In addition to MNs, a small population of eGFP-expressing interneurons (INs) was seen throughout much of the spinal cord in these transgenic mice^6,7^. With the demonstration that these INs did indeed express endogenous Hb9, they were termed Hb9 interneurons (Hb9 INs)^7^. Thus, it was found that distinct populations of spinal neurons express Hb9: MNs (somatic and SPNs) and Hb9 INs.

As Hb9 INs were shown to be glutamatergic, to be positioned in the ventromedial upper lumbar spinal cord where locomotor rhythm generation occurs^8^, and to have membrane properties that could support pacemaker-type activity, it was proposed that they could have a role in locomotor rhythm generation [^6,7^ reviewed in^9^]. To test this hypothesis, it should be possible to study locomotor activity following genetic functional removal of Hb9 INs from spinal circuits, for example using a binary strategy to eliminate vGluT2 in neurons that express Hb9. A similar genetic strategy has been used, for example, to determine a role for spinal dI3 INs^10^.

But there are several problems and potential pitfalls with such an approach. Firstly, it was noted that in Hb9::eGFP transgenic mice, there is GFP expression beyond Hb9 INs and MNs; that is, eGFP expression did not represent “true” post-natal Hb9 expression. Furthermore, Cre expression in Hb9^cre^ mice is not limited to Hb9 INs and MNs, but is expressed in widespread populations including those that expressed Hb9 transiently early in development^11^. Ergo, excision of vGluT2 using Hb9^cre^ mice would not be limited to Hb9-expressing neuronal populations, making interpretation of results using that strategy problematic.

To address this concern, we generated a novel inducible Cre mouse line, Hb9::CreER^T2^, using a BAC transgene. Our goal was to determine whether Hb9 INs play a discernable role in overground locomotion. We first characterised the pattern of tamoxifen inducible recombination and demonstrated the specificity and sensitivity of recombination in Hb9-expressing MNs and Hb9 INs. Next, we crossed this line with a vGluT2^fl/fl^ line^10,12^ to eliminate vGluT2 expression in Hb9-expressing neurons (and called the line Hb9-vGluT2^OFF^). We then demonstrated that glutamatergic transmission by Hb9 INs does not contribute to treadmill locomotion of varying speeds. We conclude that the role, if any, of Hb9 INs thus remains obscure.

## Results

### Recombination in Hb9::CreER mice is restricted to Hb9-expressing cells

**Figure 1:**
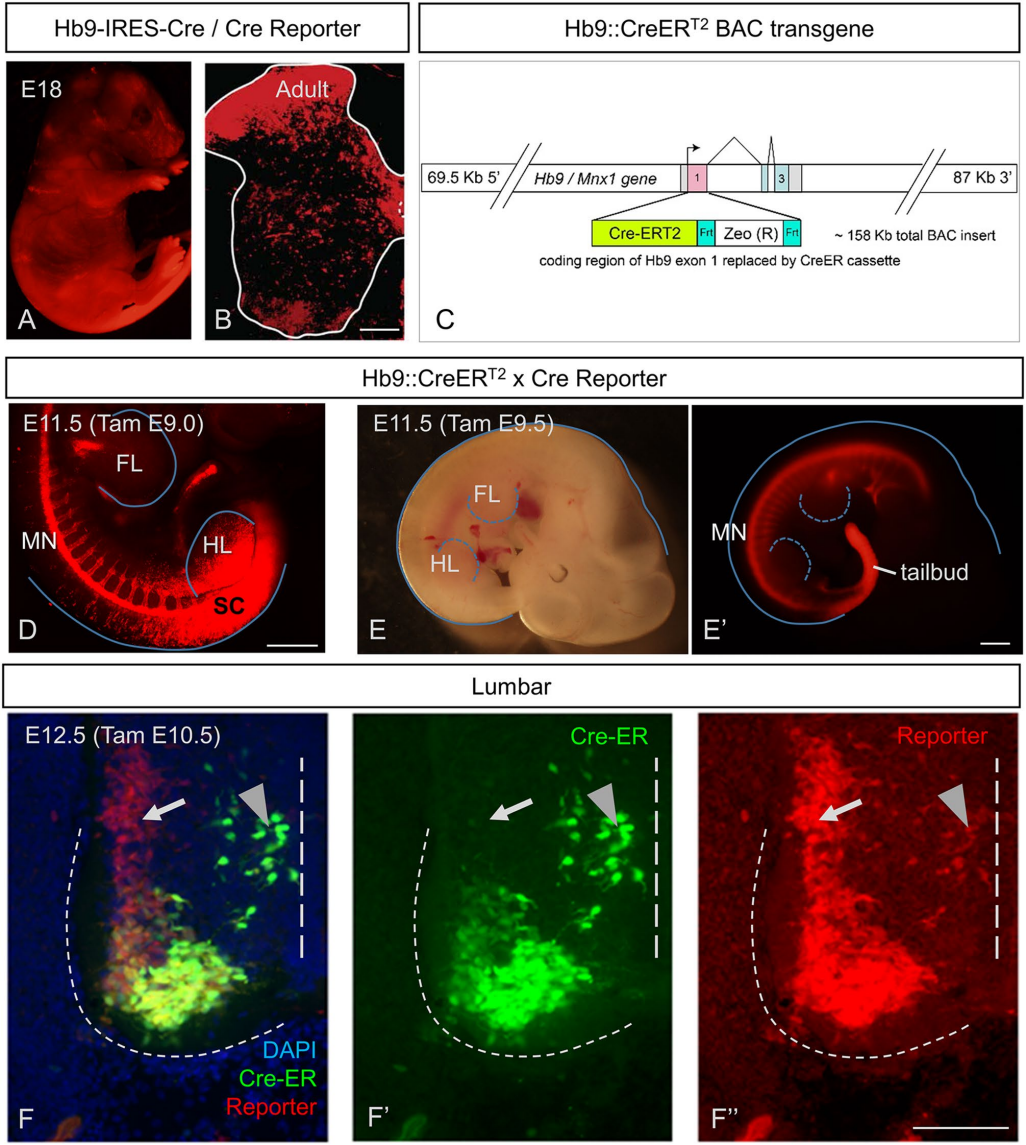
Temporal dependence of Hb9 driven CreER activity on specificity of recombination in embryonic spinal cord. (**A**,**B**) Extensive recombination of Hb9-Cre knock-in mice in many regions, shown in (**A**) E18 fetus, and (**B**) transverse postnatal spinal cord. (**C**) Diagram of the BAC transgene used to drive CreERT2 expression in Hb9^ON^ cells. (**D**–**F**) Transgenic HB9-CreER^T2^ mice efficiently and specifically enable recombination of Ai14 (tdTom) reporter in HB9^ON^ cells in the embryo, including spinal motor neurons. (**D**) Tamoxifen at E9.0 fails to confer MN specific recombination at hindlimb (HL) levels in the spinal cord (SC). (**E**) Tamoxifen at E9.5 no longer results in broad SC recombination at hindlimb levels and has activity restricted to the MN column. (**F**) Early activation by tamoxifen at E10.5 labels the MN that by E12.5 have downregulated Hb9 (arrow), as reflected by antibody staining for Cre to identify current sites of Cre-ER expression. Arrowhead indicates nascent MN that are still migrating from the midline, but may also include Hb9 INs. Scale bars: (**B**) and (**F**) 100 µm; (**D**,**E**) 500 µm.

Like others^11^, we initially used an Hb9^cre^ mouse line, but in early experience, found widespread expression of Cre-reporter throughout the embryo (Fig. 1A), all levels of the spinal cord, and in neurons throughout all laminae in the spinal cord (Fig. 1B; noted over many mice, but n = 2 formally analyzed for this study). It was thus clear that we should abandon this strategy.

We therefore developed an inducible Hb9::CreER^T2^ mouse line (Fig. 1C; hereafter called Hb9::CreER), and proceeded to study the selectivity of recombination following administration of tamoxifen (TAM) initially between E9 and E14 in Hb9::CreER;Rosa26-lox-stop-lox-tdTomato (together Hb9::CreER;tdTom). Expression of tdTom was evident as early as 24 h following TAM. At early embryonic stages (E9; Fig. 1D), TAM led to widespread expression of tdTom in the spinal cord, presumably due to early expression of Hb9 outside of the MN lineage in the caudal neural plate at this time point (Fig. 1D). This result is similar to the pattern of expression seen in Hb9^cre^;tdTom mice (cf. Fig. 1A).

On the other hand, administration of TAM even 12 h later (E9.5) led to more restricted expression patterns, with clear specificity of recombination (Fig. 1E). In particular, ventral horn neurons in the region of motor pools expressed the reporter along with various other neurons (Fig. 1F). But it was evident that there were two clusters of tdTom^ON^ MNs, one which maintained Cre-ER expression at E12.5, and one which had very low Cre-ER protein levels (Fig. 1F, arrow). These clusters corresponded to two distinct motor columns, the lateral and medial portions of the lateral motor columns (LMC-L and LMC-M), respectively. The populations of somatic MNs that showed recombination dependent expression of tdTom depended on the timing of TAM. While the lateral aspect of the lateral motor column (LMC-L) expressed tdTom with administration any time after E9.5, the medial portion (LMC-M) only expressed tdTom if TAM was given prior to E11.5. This downregulation of Hb9::CreER is consistent with previous observations that endogenous Hb9 expression is down-regulated in LMC-M motor neurons by E12.5^13,14^. Note also that motor neurons that have not yet settled in lamina IX express CreER at this embryonic stage (Fig. 1F, arrowhead). Thus, embryonic administration of TAM in Hb9::CreER mice leads to specific activation of Cre recombinase, accurately marking Hb9-expressing motor neurons (Hb9^ON^ MN) in time and space.

**Figure 2:**
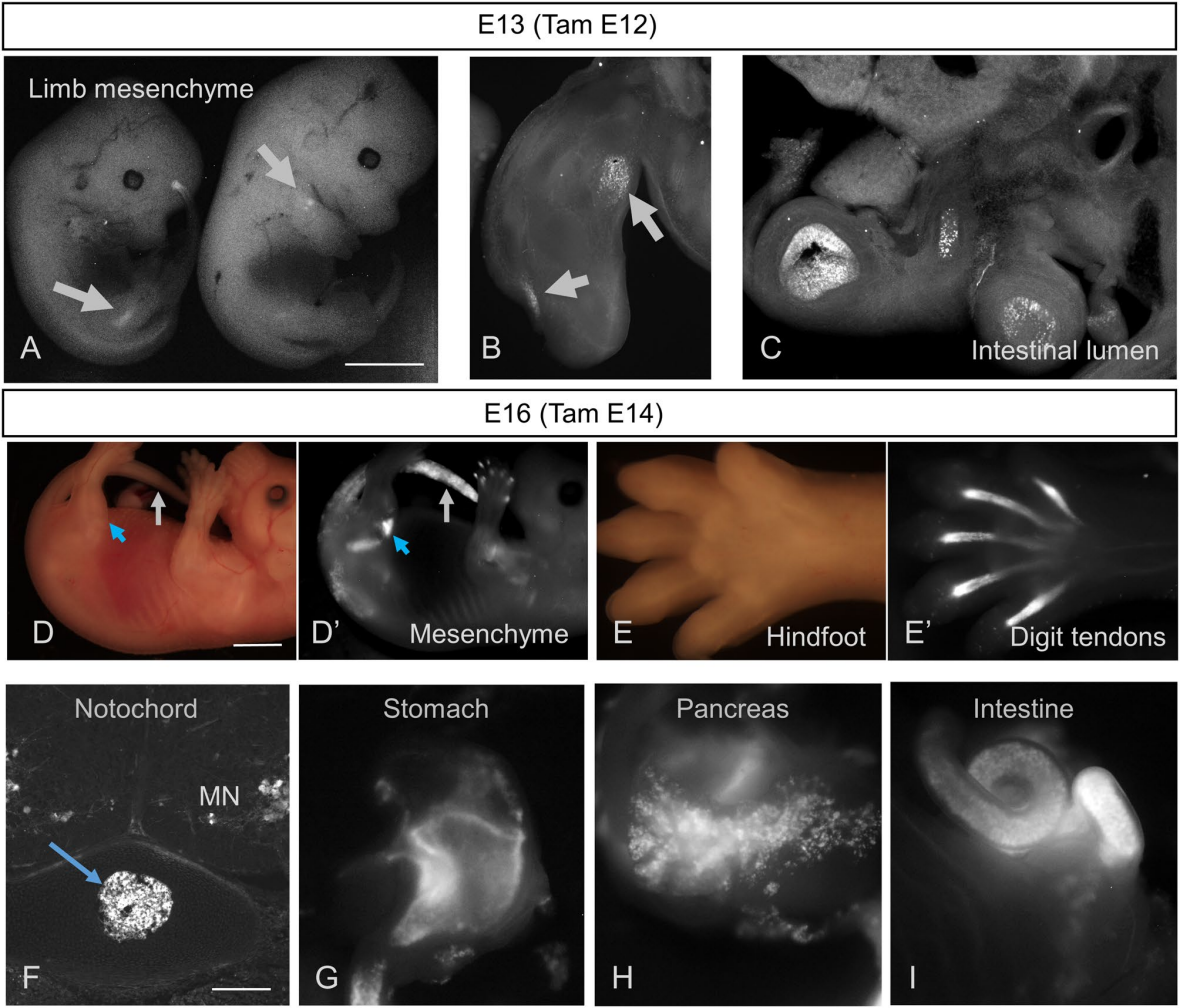
Sites of pre-natal Hb9-CreER recombination outside of the CNS. (**A**–**C**) ROSA-lox-stop-lox-EYFP reporter expression in mid-gestation HB9-CreER mice following IP administration of tamoxifen to pregnant dam at E12, revealing recombination outside of the CNS evident in limbs of E13 animals (**A**), vibratome sections of forelimb (**B**), and the lumen of the intestine (**C**). (**D**,**E**) Brightfield (**D**,**E**) versus Ai14 tdTomato reporter expression (**D′**,**E′**) in E16 mice induced with tamoxifen at E14, showing recombination in limb and tailbud mesenchyme (**D′**) and tendons of the knee (**D′**, blue arrow) and Hindfoot (**E′**). (**F**–**I**) Additional sites of Ai14 tdTomato reporter expression after E14 tamoxifen induction, including known sites of non-neuronal Hb9 expression such as notochord (**F**), stomach (**G**), pancreas (**H**), and intestine (**I**). Scale bars: (**A**) and (**D**), 2 mm; (**F**) 250 µm.

Tamoxifen administered at E12—when MNs are specifically labeled within the spinal cord—also led to reporter expression outside the central nervous system (Fig. 2A–C). Delaying TAM administration until E14 did not alter this non-neuronal recombination (Fig. 2D–I). Sites of recombination included forelimb and hindlimb mesenchyme (Fig. 2A,B,D′), which may correspond to Hb9 expression in developing cartilage (Fig. 2D′,E′); gastrointestinal lumen (Fig. 2C,I); digit tendons (Fig. 2E); notochord (Fig. 2F); stomach (Fig. 2G); and pancreas (Fig. 2H). Of note, there was no evidence of tdTom expression in sensory neurons: dorsal root ganglia had no reporter expression.

**Figure 3:**
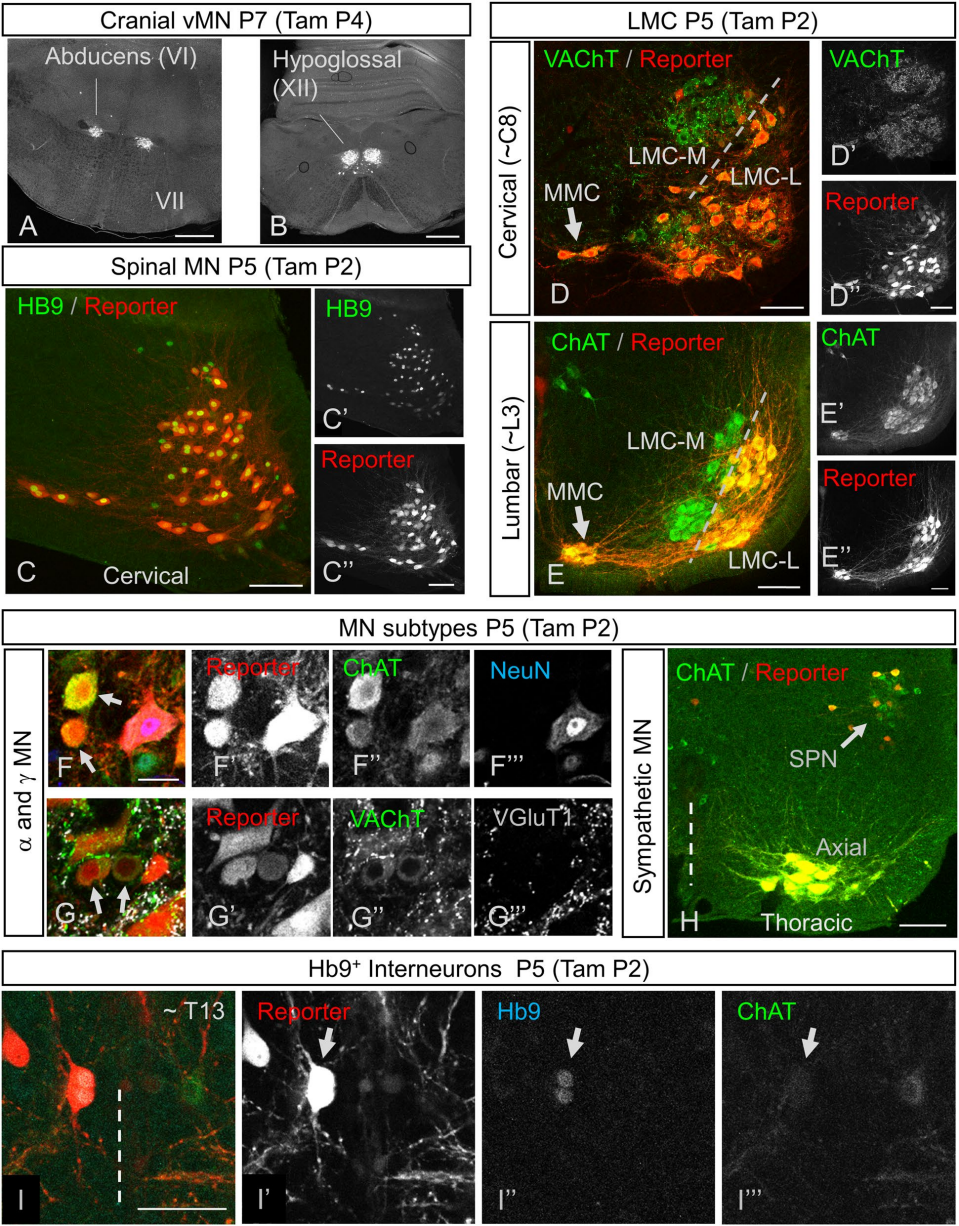
Postnatal tamoxifen induced recombination specific to Hb9^ON^ neurons. (**A**,**B**) Ai6 Cre-reporter expression in vMN cranial nuclei following P4 Tamoxifen administration to Hb9::CreER mice, showing specific recombination in motor nuclei VI (**A**) and XII (**B**) but no other brainstem MN such as VII (**A**). (**C**) In spinal cord, tamoxifen administration at P2 results in high correspondence between recombination in Ai14 reporter (**C**,**C′**) and endogenous Hb9 protein expression. (**D**,**E**) Consistent with Hb9 expression patterns, post-natal Hb9::CreER recombination distinguishes LMC medial and lateral sub-divisions at both cervical (**D**) and lumbar (**E**) levels, as revealed by Ai14 reporter pattern in all MN visualized with either VAChT (**D**) or ChAT (**E**), following tamoxifen induction at P2. (**F**–**G**) Both α- and γ- MNs undergo efficient recombination in Hb9-CreER mice following tamoxifen administration at P2. α-MN are identified as larger MN that are NeuN^ON^ (**F**), or with dense VGluT1 and VAChT puncta on their soma (**G**). γ-MN are smaller MN that are NeuN^OFF^ (arrows in F; scale bar = 25 µm), or with little to no VGluT1 and VAChT puncta on their soma. (**H**) Sympathetic Preganglionic Neurons (SPNs) at thoracic levels of spinal cord undergo recombination in Hb9::CreER mice following tamoxifen administration at P2. (**I**) Hb9 INs in Hb9::CreER mice efficiently undergo inducible recombination following tamoxifen at P2 (Level T13–L1 shown). Hb9 INs (arrow) in a single optical section are identified by size and position near the central canal, expression of Hb9 protein (**I**,**I″**) and lack of ChAT. Scale bars: (**A**,**B**) 500 µm; (**C**–**E**) and (**H**) 100 µm; (**F**) (also applies to **G**) and (**I**) 50 µm.

We next turned to postnatal (up to P7) TAM, which also resulted in efficient recombination in Hb9^ON^ motor neurons: reporter expression in brain stem MNs was specific to Hb9-expressing ventral MNs (vMNs), including abducens (Fig. 3A) and hypoglossal (Fig. 3B) nuclei. We noted no other supraspinal expression of reporter except in the choroid plexus (note, for example, lack of expression in the facial nucleus (VII), which is of branchial origin, Fig. 3A). In spinal MNs, postnatal tamoxifen induced highly efficient recombination in Hb9^ON^ MNs (Fig. 3C). During development, LMC-M motor neurons down-regulate Hb9 (see above), and correspondingly these MNs, identified by their location and expression of cholinergic markers VAChT (Fig. 3D) or ChAT (Fig. 3E), were not labelled with tdTom. However, in Hb9-expressing populations such as the medial motor column (MMC) and LMC-L motor neurons, recombination was efficient, with almost all motor neurons undergoing recombination. In addition, sympathetic preganglionic neurons (SPNs) express Hb9^4^, and they too showed recombination following TAM (Fig. 3H) (Note that, consistent with patterns of Hb9 expression, other cholinergic neurons did not).

We next examined Hb9::CreER post-natal recombination in α- and γ-MN subtypes, as non-BAC based Hb9::GFP transgenic mice display selective expression in α-MN^15^. We identified γ-MNs as small, ChAT^ON^ neurons with a visible nucleus that did not express NeuN (Fig. 3F), or those that had a paucity of primary afferent (labelled by vGluT1) or C-bouton (labelled by vAChT) inputs (Fig. 3G). Using postnatal TAM regimens, we identified efficient recombination in both NeuN^OFF^, VGluT1^low^_,_ VAChT^low^ γ-MNs as well as NeuN^ON^, VGluT1^high^, VAChT^high^ α-MNs (Fig. 3F,G). Therefore, within Hb9^ON^ motor columns the Hb9::CreER BAC line efficiently recombines in all MN subtypes.

We next turned our attention to Hb9 interneurons: a population of glutamatergic neurons in medial lamina VIII above the second lumbar segment (L2)^7^. Following postnatal TAM administration, there was CreER activation in this region as noted by reporter expression (Fig. 3I). These neurons were positive for Hb9 and negative for ChAT, indicating that these are Hb9 INs (Fig. 3I). Virtually all visually-identified Hb9 INs, identified by their location, size, and Hb9 expression, expressed the reporter (see β-gal expression below for quantification).

Together, these data reflect the specificity of our approach, in which recombination is seen primarily during the times when, and in the neurons in which, endogenous Hb9 is expressed. That is, recombination in Hb9::CreER mice induced by TAM any time after E10.5 and including the early post-natal period was seen in MNs that endogenously express Hb9: LMC-L MNs, MMC MNs, SPNs, vMN brain stem MNs, and Hb9 INs. But when TAM was administered prior to E11.5, there was also recombination in LMC-M MNs (Fig. 1F). Administration prior to E9.5 led to widespread, non-specific recombination in caudal regions (Fig. 1D). These data indicate that these inducible Hb9::CreER mice can be used for spatially and temporally specific and sensitive recombination strategies aimed at Hb9-expressing MNs and Hb9 INs.

### vGlut2-dependent transmission by Hb9 INs does not affect treadmill locomotion

**Figure 4:**
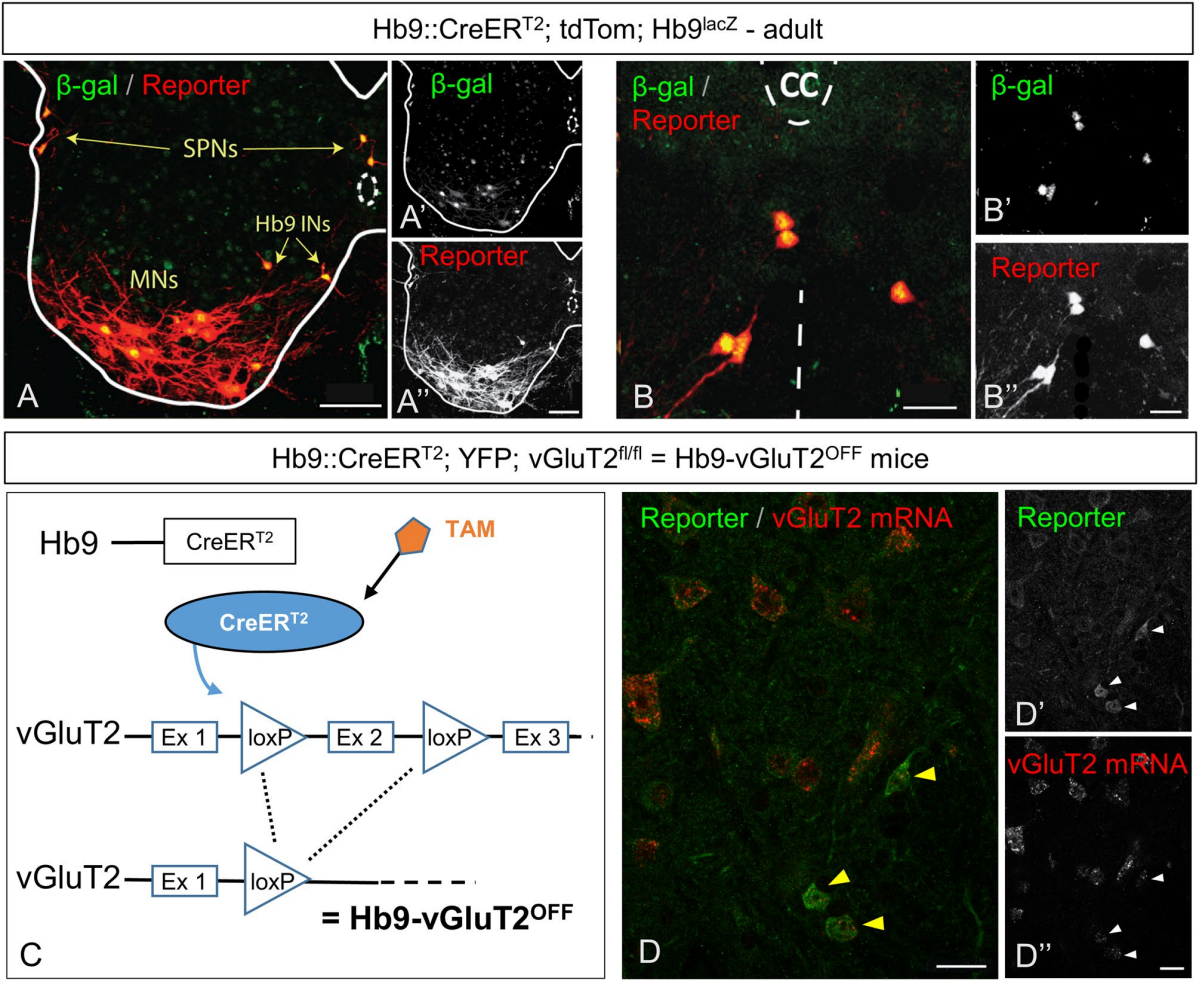
Conditional recombination in adult Hb9::CreER mice. (**A**) Following P5-P7 tamoxifen administration to Hb9^lacZ^;Hb9::CreER;R26-lox-stop-lox-tdTom mice, there is overlap of expression of β-gal and td-Tom reporter in specific neurons: SPNs, MNs, and Hb9 INs. (**B**) Higher magnification image of Hb9 INs in the ventromedial region of upper lumbar spinal segment. (**C**) Illustration of conditional CreER-mediated excision of vGluT2 in Hb9 INs following tamoxifen administration, creating Hb9-vGluT2^OFF^ mice (TAM: tamoxifen, Ex: exon). (**D**) Fluorescent in situ hybridization showing elimination of vGluT2 mRNA in Hb9::CreER; R26-lox-stop-lox-YFP; vGluT2^fl/fl^ mice. Hb9 INs (arrowheads) lack vGluT2 mRNA following recombination. Single optical section. Scale bar: (**A**) 50 µm; (**B**) and (**D**) 20 µm.

As our goal was to study mature mice, we next ensured that expression induced by postnatal TAM administration led to persistent and specific expression in adult mice (Fig. 4A). When crossed into Hb9^lacZ^ knock-in mice^16^, reporter expression in the adult was 95% concordant with β-gal expression (205/216 tdTom neurons in 3 mice also expressed β-gal), and was evident in MNs, SPNs, and Hb9 INs (Fig. 4A,B). We did not find β-gal-expressing neurons that did not express tdTom. That is, recombination was efficient at this age, and there was no evidence of substantial off-target recombination.

Given that Hb9 interneurons are glutamatergic and express vGluT2^7^ (and not vGluT1 or vGluT3^17^), whereas the primary neurotransmitter of MNs is acetylcholine, we reasoned that we could functionally remove Hb9 INs from circuits by crossing Hb9::CreER mice with vGluT2^flox/flox^ mice^12^, to yield Hb9-vGluT2^OFF^ mice (Fig. 4C), in which glutamatergic transmission from neurons expressing Hb9 would be eliminated^10^. This floxed vGluT2 mouse strain has been used with numerous Cre drivers where it has been shown to be effective^10–12^. Furthermore, in situ hybridisation for vGluT2 mRNA in Hb9-vGluT2^OFF^ mice confirmed that this strategy was effective (n = 2; Fig. 4D; cf. Figure 3A in^7^).

**Figure 5:**
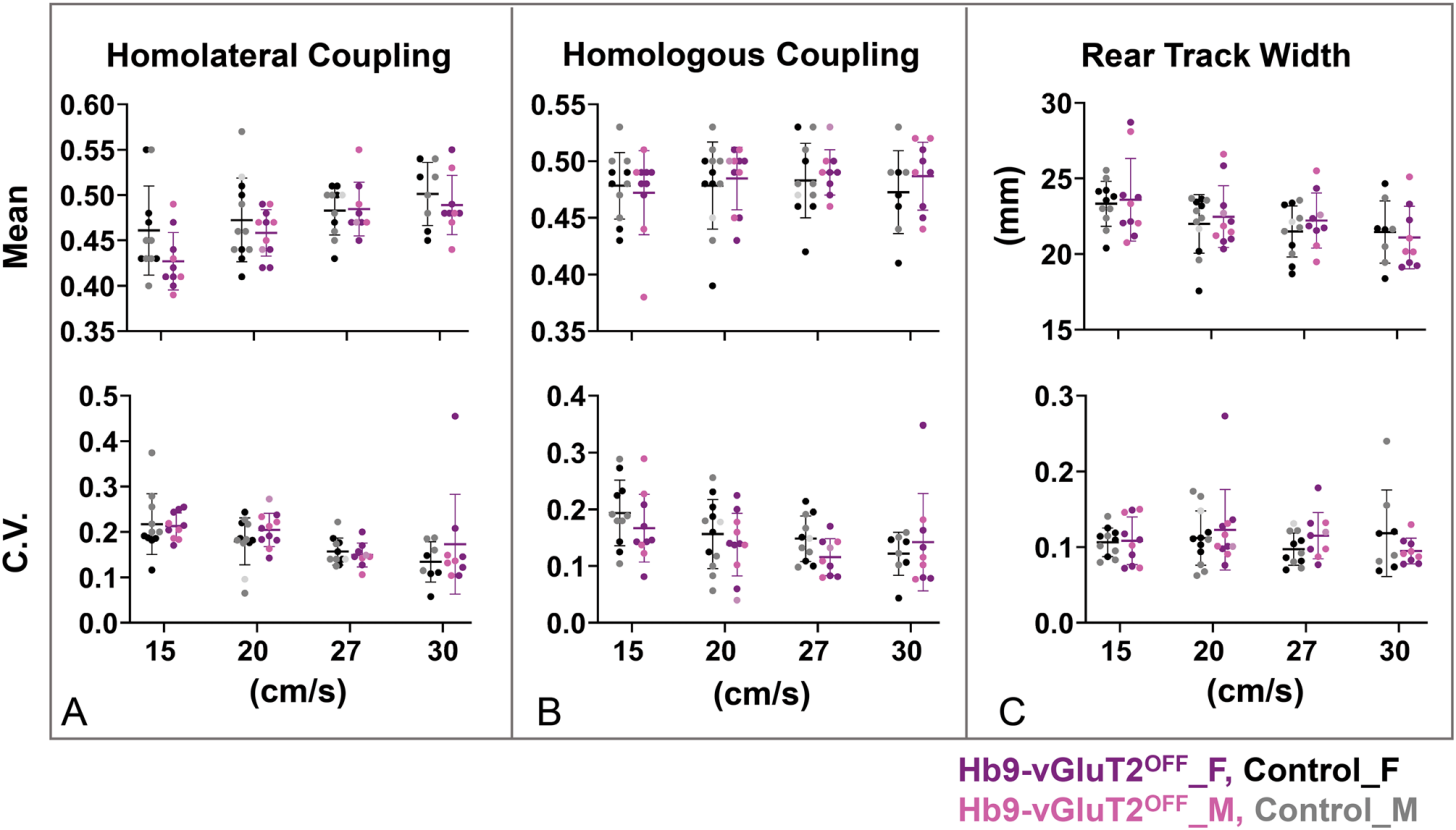
The pattern of locomotion in Hb9-vGluT2^OFF^ mice is similar to that of controls. (**A**) No significant difference was observed in homolateral coupling compared to controls (upper panel). There were no differences in the coefficients of variation (lower panel), except for difference between females and males in the control group at 15 cm/s (P = 0.04, Table 3). (**B**) Similarly, neither homologous coupling (upper panel) nor its coefficients of variation (lower panel) were different. (**C**) Rear track widths were also no different (upper panel), with similar coefficients of variation (lower panel). Numbers of mice used: total of 12 control mice, with n = 11 (5 females), 12 (5 females + 1 not specified), 11 (5 females + 1 not specified), and 8 (4 females) for 15, 20, 27, and 30 cm/s, respectively; and total of 11 Hb9-vGluT2^OFF^ mice: n = 10 (6 females), 11 (6 females + 1 not specified), 9 (5 females + 1 not specified), and 9 (5 females), for the 4 speeds, respectively. Details of statistics are in Tables 1, 2, and 3. Black: Control Female; Gray: Control Male; Light grey: Control sex not specified; Purple: Hb9-vGluT2^OFF^ Female; Pink: Hb9-vGluT2^OFF^ Male; Lavender: Hb9-vGluT2^OFF^ sex not specified.

**Figure 6:**
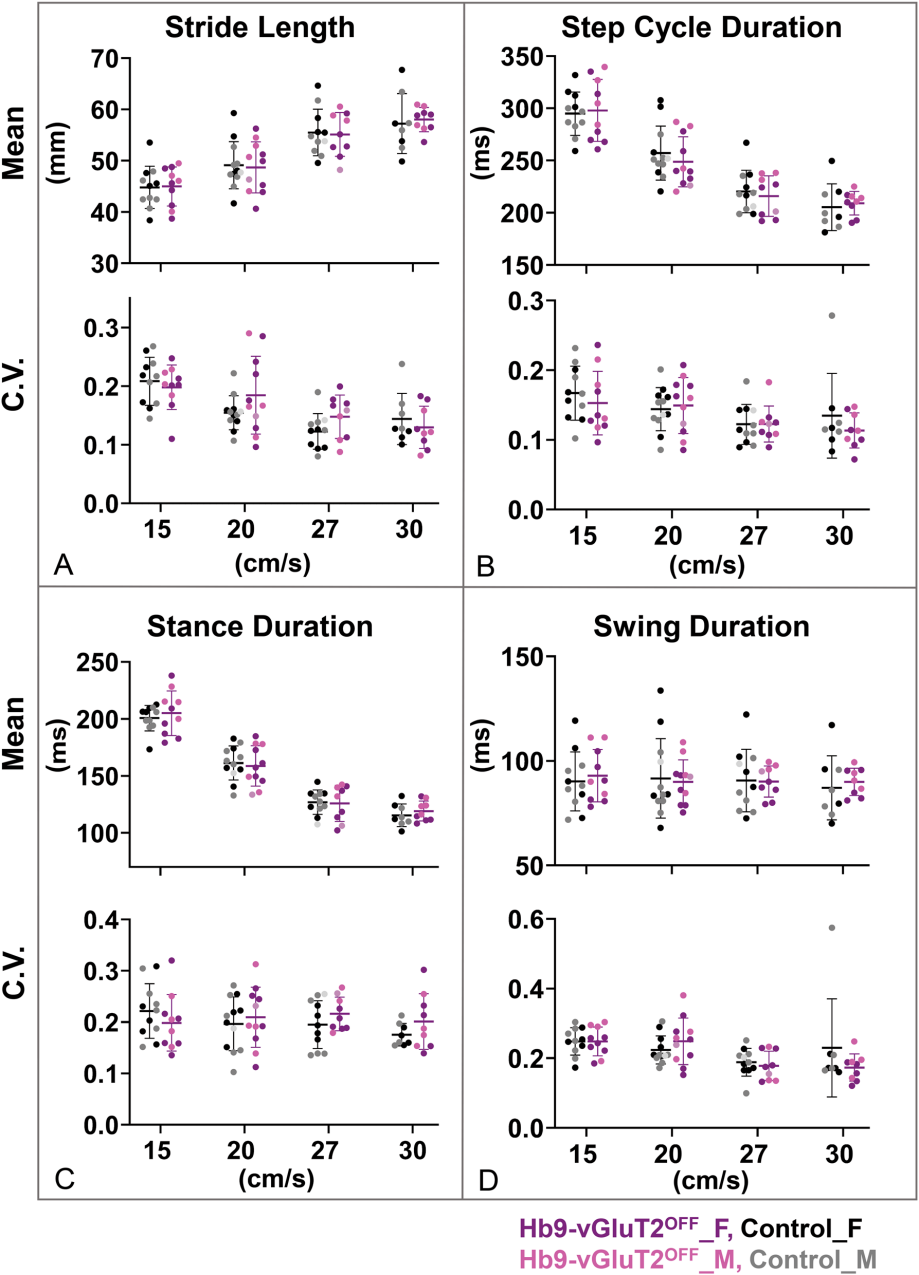
The timing of locomotion in Hb9-vGluT2^OFF^ mice is similar to that of controls. (**A**) Stride lengths (upper panel) and their coefficients of variation (lower panel) were the same in the mutant and control mice. (**B**) Similarly, no difference was observed in cycle durations (upper panel) or their coefficients of variation (lower panel). (**C**) Stance durations were also similar (upper panel), as were their coefficients of variation (lower panel). (**D**) There was no difference in swing duration (upper panel). The coefficients of variation were also the same at all speeds (lower panel). Number of mice used at the 4 speeds for the control group (total 12) were 11 (5 females), 12 (5 females + 1 not specified), 11 (5 females + 1 not specified), and 8 (4 females), and for the Hb9-vGluT2^OFF^ group (total 11) were 10 (6 females), 11 (6 females + 1 not specified), 9 (5 females + 1 not specified), and 9 (5 females). Details of statistics are in Tables 2 and 4. Black: Control Female; Gray: Control Male; Light grey: Control sex not specified; Purple: Hb9-vGluT2^OFF^ Female; Pink: Hb9-vGluT2^OFF^ Male; Lavender: Hb9-vGluT2^OFF^ sex not specified.

We next assessed these mice in the sixth post-natal week at various treadmill locomotor speeds, quantifying a number of locomotor parameters as well as their variability (Figs. 5, 6). Parameters related to the pattern of locomotion—homolateral (Fig. 5A) and homologous (Fig. 5B) coupling, and rear track width (Fig. 5C)–did not differ between the control (n = 12, of which n = 8 could maintain the highest speed) and Hb9-vGLuT2^OFF^ (n = 11, of which n = 9 could maintain the highest speed) mice. That is, while there were some differences related to speed (homolateral coupling and rear track width), there were no differences between genotypes (Table 1). Furthermore, while the variability in these parameters differed depending on speed (homolateral and homologous coupling), there was no effect of genotype on these variabilities (lower panels of Fig. 5A–C; Table 1). Finally, we analysed the data taking into consideration the sex using a one way ANOVA, and found no differences in the 4 (sex × genotype) groups, except for the variability of the homolateral coupling at the slowest speed (Table 2). Multiple comparisons demonstrated that this difference arose from a difference between females and males within the control group (P = 0.04; Table 3). That is, there were no differences between the control and Hb9-vGLuT2^OFF^ mice. These data indicate that glutamatergic transmission by Hb9 INs does not contribute to the generation of the locomotor pattern required for treadmill locomotion.

**Table 1:** Statistics for treadmill locomotion pattern parameters.

Parameter	Treadmill speed (cm/s)	Control mean	Control SD	Hb9-vGluT2OFF mean	Hb9-vGluT2OFF SD	F (DFn, DFd) speed	F (DFn, DFd) genotype	F (DFn, DFd) interaction	P value speed	P value genotype	P value interaction
Homolateral coupling average						F (3, 52) = 16.91	F (1, 21) = 1.533	F (3, 52) = 2.371	** < 0.0001**	0.2293	0.081
	15	0.46	0.05	0.43	0.03						
	20	0.47	0.05	0.46	0.03						
	27	0.48	0.03	0.48	0.03						
	30	0.50	0.03	0.49	0.03						
Homolateral coupling variability						F (3, 52) = 7.153	F (1, 21) = 1.061	F (3, 52) = 0.9638	**0.0004**	0.3148	0.4169
	15	0.22	0.07	0.21	0.03						
	20	0.18	0.05	0.20	0.04						
	27	0.16	0.03	0.15	0.03						
	30	0.13	0.04	0.17	0.11						
Homologous coupling average						F (3, 52) = 1.042	F (1, 21) = 0.2504	F (3, 52) = 0.4604	0.3818	0.622	0.7111
	15	0.48	0.03	0.47	0.04						
	20	0.48	0.04	0.48	0.03						
	27	0.48	0.03	0.49	0.02						
	30	0.47	0.04	0.49	0.03						
Homologous coupling variability						F (3, 52) = 5.482	F (1, 21) = 0.6358	F (3, 52) = 1.445	**0.0024**	0.4342	0.2404
	15	0.19	0.06	0.17	0.06						
	20	0.16	0.06	0.14	0.06						
	27	0.15	0.04	0.12	0.03						
	30	0.12	0.04	0.14	0.09						
Rear track width average (mm)						F (3, 52) = 24.85	F (1, 21) = 0.1032	F (3, 52) = 0.6940	** < 0.0001**	0.7512	0.5599
	15	23.33	1.50	23.60	2.74						
	20	21.99	1.94	22.47	2.04						
	27	21.49	1.69	22.22	1.82						
	30	21.46	2.07	21.09	2.07						
Rear track width variability						F (3, 52) = 0.6677	F (1, 21) = 0.004721	F (3, 52) = 1.882	0.5757	0.9459	0.144
	15	0.11	0.02	0.11	0.03						
	20	0.11	0.04	0.12	0.05						
	27	0.10	0.02	0.12	0.03						
	30	0.12	0.06	0.09	0.02						

Repeated measures two way ANOVA. Bold indicates significant P values. See “Methods” for details.

**Table 2:** Statistics for sex × genotype analysis for treadmill locomotion pattern and timing parameters using one way ANOVA.

Parameter	Treadmill speed (cm/s)	Control_F mean	Control_F SD	Control_M mean	Control_M SD	Hb9-vGluT2OFF_ F mean	Hb9-vGluT2OFF_ F SD	Hb9-vGluT2OFF_ M mean	Hb9-vGluT2OFF_ M SD	ANOVA F	ANOVA R-squared	Parametric (P value)
Homolateral coupling average	15	0.47	0.05	0.45	0.05	0.43	0.03	0.43	0.04	1.28	0.18	0.31
	20	0.46	0.04	0.48	0.05	0.45	0.03	0.47	0.02	0.44	0.07	0.73
	27	0.48	0.03	0.48	0.02	0.48	0.02	0.50	0.04	0.62	0.12	0.61
	30	0.49	0.04	0.51	0.03	0.50	0.03	0.48	0.04	0.50	0.10	0.69
Homolateral coupling variability	15	0.17	0.03	0.25	0.07	0.22	0.03	0.20	0.02	3.46	0.38	**0.04**
	20	0.20	0.03	0.18	0.06	0.19	0.03	0.21	0.03	0.65	0.10	0.59
	27	0.16	0.02	0.16	0.04	0.16	0.03	0.13	0.02	0.73	0.14	0.55
	30	0.11	0.04	0.16	0.03	0.21	0.14	0.13	0.02	1.22	0.22	0.34
Homolog coupling average	15	0.47	0.03	0.49	0.03	0.48	0.01	0.46	0.06	1.12	0.17	0.37
	20	0.47	0.04	0.49	0.03	0.48	0.03	0.49	0.03	0.45	0.07	0.72
	27	0.47	0.04	0.49	0.03	0.49	0.01	0.48	0.02	0.43	0.08	0.73
	30	0.46	0.03	0.49	0.04	0.48	0.03	0.49	0.04	0.86	0.17	0.48
Homologous coupling variability	15	0.20	0.06	0.19	0.06	0.15	0.04	0.19	0.08	0.85	0.13	0.48
	20	0.18	0.04	0.13	0.07	0.15	0.06	0.14	0.03	0.74	0.12	0.54
	27	0.15	0.05	0.14	0.04	0.12	0.04	0.11	0.03	1.11	0.19	0.38
	30	0.11	0.05	0.13	0.03	0.16	0.11	0.12	0.04	0.46	0.10	0.72
Rear track width average (mm)	15	23.23	1.60	23.41	1.55	23.63	2.71	23.56	3.21	0.03	0.01	0.99
	20	21.60	2.67	22.37	1.49	22.23	2.06	22.79	2.56	0.23	0.04	0.87
	27	21.01	2.22	21.84	1.29	22.37	1.14	21.85	3.22	0.42	0.08	0.74
	30	21.59	2.57	21.33	1.83	20.67	1.96	21.62	2.37	0.19	0.04	0.90
Rear track width variability	15	0.11	0.01	0.10	0.02	0.10	0.03	0.12	0.04	0.39	0.06	0.76
	20	0.11	0.01	0.11	0.05	0.14	0.07	0.11	0.02	0.43	0.07	0.74
	27	0.09	0.02	0.10	0.02	0.12	0.04	0.11	0.02	1.11	0.19	0.38
	30	0.10	0.03	0.14	0.07	0.09	0.01	0.10	0.02	1.44	0.25	0.28
Stride length average (mm)	15	46.04	5.45	43.75	2.71	44.52	3.98	45.68	4.00	0.36	0.06	0.78
	20	50.25	6.85	48.41	2.65	47.86	5.67	50.54	4.60	0.34	0.06	0.80
	27	56.71	5.46	54.62	4.26	54.12	3.24	59.06	1.38	1.12	0.19	0.37
	30	57.17	7.67	57.30	4.55	57.46	2.41	58.71	2.44	1.12	0.19	0.37
Stride length variability	15	0.21	0.04	0.21	0.04	0.19	0.05	0.21	0.02	0.29	0.05	0.83
	20	0.15	0.02	0.16	0.04	0.19	0.07	0.18	0.08	0.75	0.12	0.54
	27	0.11	0.02	0.13	0.04	0.17	0.03	0.11	0.03	3.34	0.42	0.05
	30	0.12	0.01	0.17	0.05	0.14	0.04	0.12	0.03	2.00	0.32	0.16
Cycle duration average (ms)	15	304.00	27.40	287.20	10.49	287.40	29.85	313.80	24.40	1.44	0.20	0.27
	20	265.20	38.40	251.10	13.32	246.20	18.71	258.10	32.27	0.54	0.09	0.66
	27	228.70	25.44	214.90	14.43	207.00	17.26	235.90	3.72	2.12	0.31	0.14
	30	211.70	29.82	199.00	13.52	203.30	11.38	216.40	6.08	0.86	0.17	0.49
Cycle duration variability	15	0.16	0.03	0.18	0.05	0.15	0.05	0.16	0.04	0.41	0.07	0.75
	20	0.15	0.03	0.14	0.04	0.15	0.05	0.15	0.04	0.09	0.02	0.97
	27	0.12	0.03	0.12	0.04	0.11	0.02	0.14	0.04	0.54	0.10	0.66
	30	0.11	0.03	0.16	0.08	0.10	0.03	0.12	0.02	1.16	0.21	0.36
Stance duration average (ms)	15	201.50	15.99	200.00	6.74	198.60	22.02	214.60	11.58	1.00	0.15	0.42
	20	162.00	16.59	162.20	15.95	161.90	14.69	160.30	21.34	0.01	0.00	1.00
	27	130.00	12.20	127.70	5.40	122.40	16.25	138.10	5.37	1.21	0.21	0.34
	30	117.40	13.63	113.40	6.04	115.50	9.51	123.70	5.96	0.91	0.17	0.46
Stance duration variability	15	0.22	0.06	0.23	0.05	0.20	0.07	0.20	0.04	0.31	0.05	0.82
	20	0.21	0.04	0.19	0.07	0.21	0.06	0.21	0.07	0.16	0.03	0.92
	27	0.20	0.03	0.18	0.06	0.20	0.02	0.23	0.05	0.90	0.16	0.47
	30	0.17	0.02	0.18	0.03	0.21	0.07	0.19	0.04	0.59	0.12	0.63
Swing duration average (ms)	15	94.48	18.40	86.69	9.57	88.81	10.53	99.18	14.15	0.88	0.13	0.47
	20	97.56	27.46	85.38	10.02	84.35	7.88	97.82	11.05	1.15	0.17	0.36
	27	95.81	18.37	83.79	10.53	84.59	4.68	97.80	1.80	1.70	0.27	0.21
	30	90.69	20.66	83.63	9.41	87.82	7.15	92.72	5.38	0.44	0.09	0.73
Swing duration variability	15	0.24	0.04	0.26	0.04	0.23	0.03	0.27	0.05	0.82	0.13	0.50
	20	0.24	0.03	0.21	0.05	0.23	0.07	0.28	0.08	1.05	0.16	0.40
	27	0.18	0.03	0.19	0.06	0.19	0.04	0.17	0.06	0.19	0.04	0.90
	30	0.18	0.02	0.28	0.20	0.16	0.03	0.19	0.04	1.26	0.23	0.33

Bold indicates significant P values. See “Methods” for details.

**Table 3:** Statistics for multiple comparison of sex × genotype for the homolateral coupling variability at 15 cm/s using Bonferroni correction.

Multiple comparisons for homolateral coupling varaiblity at 15 cm/s	Adjusted P value
HB9OFF_F vs. HB9OFF_M	> 0.99
HB9OFF_F vs. Control_F	0.44
HB9OFF_F vs. Control_M	> 0.99
HB9OFF_M vs. Control_F	> 0.99
HB9OFF_M vs. Control_M	0.38
Control_F vs. Control_M	**0.04**

Bold indicates significant P values. See “Methods” for details.

**Figure 7:**
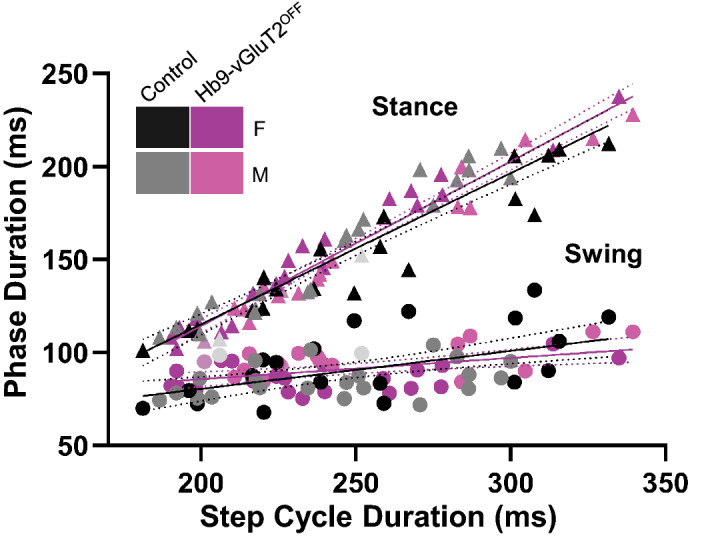
Relationships between phase duration and step cycle duration in Hb9-vGluT2^OFF^ mice were similar to controls. There was no significant difference in the relationship between stance phase and speed (Control R^2^ = 0.89, Hb9-vGluT2^OFF^ R^2^ = 0.95, P = 0.2), and no difference in swing phase in relation to speed (Control R^2^ = 0.28, Hb9-vGluT2^OFF^ R^2^ = 0.25 P = 0.15). The numbers of mice tested at treadmill speeds of 15, 20, 27, and 30 cm/s were: Control 11 (5 females), 12 (5 females + 1 not specified), 11 (5 females + 1 not specified), and 8 (4 females), respectively (total n = 12), and for Hb9-vGluT2^OFF^ mice: n = 10 (6 females), 11 (6 females + 1 not specified), 9 (5 females + 1 not specified), and 9 (5 females), respectively (total n = 11). Black: Control Female; Gray: Control Male; Light grey: Control sex not specified; Purple: Hb9-vGluT2^OFF^ Female; Pink: Hb9-vGluT2^OFF^ Male; Lavender: Hb9-vGluT2^OFF^ sex not specified.

As we and others^6,7,9,18^ had previously suggested that Hb9 INs may provide a “clock” function for locomotor circuits (see “Discussion”), we suspected there may be differences, in particular increased variability, in timing parameters [stride length (Fig. 6A), step cycle duration (Fig. 6B), stance duration (Fig. 6C), and swing duration (Fig. 6D)] in Hb9-vGluT2^OFF^ mice. While most of these parameters differed depending on speed (Table 4), there were no significant differences based on genotype. Furthermore, the coefficients of variability for each of these parameters (Fig. 6, lower panels), while in some cases speed-dependent, did not differ between the different genotypes. Analysis of the data taking into consideration the sex using a one way ANOVA failed to find differences between the 4 (sex × genotype) groups (Table 2). Finally, the relationships between step cycle phases and cycle duration (Fig. 7) were the same in Hb9-vGluT2^OFF^ and control mice. That is, the absence of glutamatergic transmission by Hb9 INs did not influence the parameters related to locomotor rhythm of mice walking on a treadmill.

**Table 4:** Statistics for treadmill locomotion timing parameters.

Parameter	Treadmill speed (cm/s)	Control mean	Control SD	Hb9-vGluT2OFF mean	Hb9-vGluT2OFF SD	F (DFn, DFd) speed	F (DFn, DFd) genotype	F (DFn, DFd) interaction	P value speed	P value genotype	P value interaction
Stride length average (mm)						F (3, 52) = 130.7	F (1, 21) = 0.01024	F (3, 52) = 0.7065	** < 0.0001**	0.9204	0.5525
	15	44.79	4.12	44.99	3.81						
	20	49.12	4.61	48.69	4.98						
	27	55.50	4.54	55.11	4.26						
	30	57.23	5.84	58.01	2.36						
Stride length variability						F (3, 52) = 20.27	F (1, 21) = 0.5751	F (3, 52) = 2.626	** < 0.0001**	0.4567	0.0601
	15	0.21	0.04	0.20	0.04						
	20	0.15	0.03	0.18	0.07						
	27	0.12	0.03	0.15	0.04						
	30	0.14	0.04	0.13	0.04						
Cycle duration average (ms)						F (3, 52) = 182.4	F (1, 21) = 0.09977	F (3, 52) = 1.081	** < 0.0001**	0.7552	0.3655
	15	294.87	20.79	297.98	29.64						
	20	257.04	25.87	248.73	24.04						
	27	220.39	20.30	215.98	19.45						
	30	205.34	22.49	209.13	11.22						
Cycle duration variability						F (3, 52) = 6.127	F (1, 21) = 0.3337	F (3, 52) = 0.6572	**0.0012**	0.5696	0.5821
	15	0.17	0.04	0.15	0.05						
	20	0.14	0.03	0.15	0.04						
	27	0.12	0.03	0.12	0.03						
	30	0.13	0.06	0.11	0.03						
Stance duration average (ms)						F (3, 52) = 353.8	F (1, 21) = 0.0004317	F (3, 52) = 0.7049	** < 0.0001**	0.9836	0.5534
	15	200.68	11.21	205.02	19.54						
	20	161.32	14.96	158.74	17.74						
	27	126.88	10.68	125.83	15.84						
	30	115.41	9.98	119.13	8.78						
Stance duration variability						F (3, 52) = 1.090	F (1, 21) = 0.3011	F (3, 52) = 2.060	0.3614	0.589	0.1168
	15	0.22	0.05	0.20	0.06						
	20	0.20	0.05	0.21	0.06						
	27	0.20	0.05	0.22	0.03						
	30	0.18	0.02	0.20	0.05						
Swing duration average (ms)						F (3, 52) = 0.8343	F (1, 21) = 0.001461	F (3, 52) = 0.7162	0.4812	0.9699	0.5468
	15	90.23	14.06	92.96	12.53						
	20	91.64	19.05	89.99	10.58						
	27	90.61	14.92	90.15	7.49						
	30	87.16	15.34	90.00	6.57						
Swing duration variability						F (3, 52) = 6.890	F (1, 21) = 0.1320	F (3, 52) = 1.647	**0.0005**	0.72	0.1898
	15	0.25	0.04	0.25	0.04						
	20	0.22	0.04	0.25	0.07						
	27	0.19	0.04	0.18	0.04						
	30	0.23	0.14	0.17	0.04						

Repeated measures two way ANOVA. Bold indicates significant P values. See “Methods” for details.

Taken together, we were unable to find evidence to support the hypothesis that glutamatergic transmission by Hb9 INs plays a significant role in treadmill locomotion.

## Discussion

Hb9 is a homeodomain protein expressed in sub-populations of somatic motor neurons and a population of ventral spinal interneurons termed Hb9 interneurons whose function is unknown. In order to gain genetic access to these neurons, we made a new BAC transgenic mouse line using a tamoxifen-inducible Cre recombinase strategy, Hb9::CreER. After demonstrating the specificity of this line, we proceeded to ask whether Hb9 INs significantly contribute to locomotor activity in adult mice. To do this, we removed glutamatergic neurotransmission from Hb9 INs using an intersectional approach to delete vGluT2 from Hb9-expressing neurons. We demonstrated that doing so does not significantly impact treadmill locomotion.

### Use of Hb9::CreER mice to study motor neurons

Several methods have been used to gain genetic access to somatic motor neurons, but all—including ours—have caveats. The mouse line that we describe here can be used to study and manipulate LMC-L MNs, or with precise timing of TAM administration at E9, possibly even all MNs. While the limitation to LMC-L would be disadvantageous for many studies, it could provide the ability to compare affected (LMC-L) vs unaffected (LMC-M) motor neurons in the same animal with any given genetic manipulation. This could be useful, for example, in ALS studies^19^.

In contrast to our line, Hb9-ires-Cre knock-in mice (Mnx1^tm4(cre)Tmj^, termed Hb9^cre^ here) have been used in several studies to genetically manipulate cervical motor neurons^20,21^. To ensure specificity, these investigators used an intersectional approach. Specifically, Hb9-cre;Isl2-lox-stop-lox-DTA (or DTX) crosses were used to eliminate most cervical motor neurons^20,21^. Of note, the focus in those studies was the neuromuscular junction. This intersectional approach would not affect neuronal populations other than motor neurons as isl2-expressing INs do not express Hb9^21^. Hb9-driven Cre expression was not extensively investigated in these studies, but appeared to be confined to lamina IX (motor neurons) in a section of cervical spinal cord; this may have led others to the erroneous assumption that Hb9-ires-Cre was motor neuron specific throughout the spinal cord (see http://www.informatics.jax.org/reference/allele/MGI:2447793?typeFilter=Literature). Several years later, Cre-mediated recombination was studied in these Hb9^cre^ mice, and a gradient of non-MN recombination was evident: there was reporter expression in relatively few cells outside motor pools in the cervical cord, but extensive expression throughout the lumbar cord^22–24^. That is, there is expression of Cre beyond MN pools in these mice, and this “ectopic” expression increases from rostral to caudal. This pattern likely results from early Hb9 expression in the caudal neural plate and tail bud area, where all cell progeny derived from these neural plate progenitor cells inherit Cre-recombined DNA. Moreover, this line shows expression in supraspinal regions. Although other genetic approaches enable Cre-mediated recombination in motor neurons (Olig2^cre^or ChAT^cre^), both Olig2 and ChAT are expressed in other populations of spinal and supraspinal neurons (e.g.^25^). Further, neither can be induced in the post-natal period to study motor neurons (TAM needs to be given embryonically in Olig2-CreER mice, and in ChAT-CreER mice, expression is not confined to motor neurons^26^). Thus, the Hb9::CreER line provides improved genetic access to non-LMC-M spinal motor neurons, as well as other Hb9-expressing neurons including SPNs and Hb9 INs.

### Hb9 INs and locomotion

Our primary goal in this study was to test our long-standing hypothesis that Hb9 INs are involved in locomotion. Based on Sutton’s Law that states that one should “go for the money”^27^, we elected to test this hypothesis in treadmill locomotion, because if no effects could be observed, circuit analysis in reduced preparations would be unnecessary.

Hb9 INs were identified almost 2 decades ago, and their rhythmic, conditional bursting properties along with their location in the ventromedial upper lumbar spinal cord^3^ led to the suggestion that they perform a “clock” role to maintain the rhythm of locomotion^7,9^. Since that time, these neurons have been studied in a number of additional labs as well^18,28,29^. Some investigators have cast doubt on a role for Hb9 INs in locomotion^11^, and others concluded that, given that Hb9 INs are active following the onset of motoneuron activity, they are unlikely to be solely responsible for rhythm generation^29^. Their function in behavior has therefore remained opaque, and a putative role in rhythm generation continues to be discussed—for recent reviews, see^30,31^.

Using the Hb9^cre^ knock-in line, it was suggested that eliminating glutamatergic transmission from “Hb9::Cre-derived” neurons (vide supra) impacted locomotor rhythm in isolated neonatal spinal cord preparations^11^. But as these authors discussed, it is difficult to draw conclusions from this approach about the role of Hb9 INs, as early expression of Hb9 leads to Cre-mediated recombination in many neurons through the spinal cord, i.e. Cre recombination is too widespread and Hb9 INs too few in number relative to Cre expression to draw solid conclusions^11^. Thus, to allow more specific genetic access, it was necessary first to make a new mouse that could obviate the problems resulting from early caudal Hb9 expression. Using an inducible CreER system, we indeed found specific and sensitive recombination in Hb9 expressing neurons when activated with appropriate timing of TAM administration.

With this new mouse, we generated Hb9-vGluT2^OFF^ mice by multi-generational breeding with vGluT2^flox^ mice, which we and others have used successfully before^3,10,12^, and used these mice to quantify treadmill locomotion in the absence of glutamatergic transmission from Hb9 INs. The pattern and timing of treadmill locomotion were the same in mutant and control mice. While a statistical difference was seen in the variability of homolateral coupling at the slowest speed, multiple comparison analysis revealed that this difference was between females and males of the control group (Fig. 5A; Table 3; P = 0.04), and was not due to genotype.

It is important to note, however, that these data do not completely exclude Hb9 INs from participating in locomotion. For example, our protocol involved post-natal TAM administration, and Hb9 INs could be important during embryonic development. More interestingly, however, Hb9 INs are electrotonically coupled to other types of neurons^16^; electrotonic transmission can play an important role in synchronicity and rhythmogenesis in other systems (e.g.^32–34^). The Hb9::CreER mouse described here could be used to try to sort this out, for example by post-natally eliminating Cx36 from Hb9-expressing neurons, when motor neuron expression of Cx36 is already decreasing^35^. Selectively eliminating Hb9 INs using, for example, genetic expression of diphtheria toxin fragment A^36^ driven by the promoter for vGluT2 could also be used.

It is possible that glutamatergic transmission by Hb9 INs is involved in intrinsic rhythm generation, such as that which can be studied in isolated spinal cord preparations. We elected to test their role in treadmill locomotion, during which rhythmic sensory inputs from the limbs can entrain locomotion^37^ and affect swing and stance vs cycle time relationships^38^. That is, sensory inputs may minimize any deficits from eliminating a central rhythmogenic network. Furthermore, “adjustments” via supraspinal inputs could also be involved.

In recent years, other neurons, in particular Shox2 INs, have been identified as strong candidates for locomotor rhythmogenesis^34^. Whether there are identifiable pacemaker neurons that are necessary for locomotor rhythm remains to be seen: this question has been difficult to answer in the respiratory system, where there have been discussions over decades about whether the rhythm is generated by pacemaker neurons and/or circuit mechanisms, with no single neuronal substrate found^39–41^. For locomotion, it is interesting to consider that two populations of neurons that reciprocally inhibit each other are a prime substrate for rhythm generation^42^, and that locomotor circuits have many places for such pairs of direct or indirect reciprocal inhibition (governing movement across joints, between joints, and between limbs, for example^43^). Thus there are many microcircuits in which rhythm could be generated with or without neuronal pacemaker neurons. In retrospect, perhaps, it would have been a surprise to see profound changes in the rhythm of treadmill locomotion when removing chemical transmission from this one neuron type—Hb9 INs—from spinal cord circuits.

## Methods

### Animals

All experimental procedures at Dalhousie University were approved by the University Committee on Laboratory Animals and were in accordance with the Canadian Council on Animal Care guidelines and those procedures at Columbia University were approved in accordance with IACUC guidelines. The study was carried out in compliance with ARRIVE guidelines (Table S1).

Hb9^lacZ/+^^4^, Hb9^cre/+^^4^, and vGluT2^flox/flox^^12^ have been previously described. Rosa26-lox-stop-lox-reporters (tdTomato (Ai14, Jax#007908); EYFP (Jax#006148); or ZsGreen (Ai6; Jax#007906)) were obtained from the Jackson Laboratory.

### Hb9::CreER^T2^ DNA recombineering

The bacterial artificial chromosome (BAC) clone RP24-351I23 (CHORI BACPAC resources) containing the mouse *Mnx1* (Hb9) gene was modified using recombineering bacterial strains (NCI, http://web.ncifcrf.gov/research/brb/recombineeringInformation.aspx). The BAC was modified to remove the loxP and loxP511 sites in the pTARBAC vector backbone using homologous targeting cassettes containing ampicillin and spectinomycin resistance, respectively. A shuttle vector containing short homology arms to desired recombination sites was used to introduce CreER^T2^-BGH polyA and a Frt-Zeo-Frt selection cassette into Hb9 exon 1, replacing the Hb9 coding region. In the modified BAC, the CreER^T2^ cassette^44^ was flanked by 69.5 kb of 5′ DNA and 87 kb of 3′ DNA. The Frt-Zeo-Frt cassette is retained in the transgene but lacks a eukaryotic promoter.

Both Dalhousie University and UCL have material transfer agreements with both Columbia University, where the mice were engineered, and Novartis Forschungsstiftung, Zweigniederlassung, Friedrich Miescher Institute for Biomedical Research (“FMI”), for the CreER^T2^.

### Transgenic mouse generation

Modified BAC DNA was digested by Not1 to remove pTARBAC vector, run out on low melting point agarose, the insert gel purified, and recovered by beta-agarase digestion. DNA was dialyzed into injection buffer and oocyte injections were done in the transgenic core at the Irving Cancer Center, Columbia University Medical Center. Three Hb9::CreER founder lines (4, 8, and 10) on B6CBA hybrid backgrounds were confirmed for tamoxifen induced recombination in motor neurons between E11–E14 using a ROSA26-lox-stop-lox-EYFP reporter. Line 10 gave low level inducible activity and was terminated. Line 4 and Line 8 displayed similar inducible recombination activity in the desired cell populations and were maintained on C57BL/6J backgrounds. Both lines are viable if bred to homozygosity, but were maintained as heterozygotes. Because the lines were indistinguishable, we have used line 8 for the majority of studies described herein.

### Tamoxifen administration

Tamoxifen (Sigma T-5648) was dissolved to 20 mg/mL in 90% sesame oil/ 10% Ethanol, briefly heated to 50 °C to facilitate dissolution, and stored in frozen aliquots. For administration, stock aliquots were warmed to 37 °C for 10 min prior to injection. Pregnancies were timed by vaginal plugs, and pregnant dams (~ 30 g bodyweight) were intraperitoneally (IP) injected a single time with 0.1 mL stock (2 mg) at indicated ages. In our preliminary characterization of the mice using the ROSA26::lox-stop-lox-EYFP reporter, we found that a single intraperitoneal injection administered between E10 and E16 of tamoxifen into a pregnant female Hb9::CreER mouse was highly efficient at inducing recombination in embryonic spinal motor neurons.

In postnatal experiments, pups of both sexes (ages P2–P7) were injected subcutaneously above the dorsal neck fat pad with 0.1 mL (2 mg) stock solutions and maintained on a 37 °C heating pad for 1 h before returning to their nursing cage. Due to leakage from the subcutaneous injection site, the exact dose in pups was likely variable. We did not observe any toxicity when tamoxifen was administered by these approaches.

### Tissue preparation and labeling

Under deep ketamine/xylazine anesthesia, mice were perfused with 4% paraformaldehyde (PFA) in 0.2 M PB. Following perfusion, the spinal cords were extracted and post fixed in PFA at 4 °C overnight, then transferred to a 30% sucrose solution for 24–48 h before sectioning.

Spinal cord blocks were sectioned (50–70 μm) using a vibrating microtome and processed for immunohistochemistry on the same day, or stored in glycerol at − 20 °C for later use.

Floating sections were washed in PBS for 10 min before incubation in 50% ethanol for 30 min to enhance antibody (Ab) penetration. Sections were washed with double salt PBS (dsPBS) 3 × 10 min and transferred to blocking solution containing 10% donkey serum in 0.3 M Triton X and PBS solution (PBST) for 30 min at room temperature.

Tissues were transferred to primary Ab (Anti-DsRed-Rabbit, Rockland antibodies and assays PA, USA 1:2000; Anti-β-gal Goat or Mouse, Santa Cruz Biotechnology, Inc, Dallas, Tx, USA 1:500; Anti-GFP-Sheep or Mouse, GenWay Biotech, Inc. San Diego, CA, USA and Novus Biological inc. Littleton, CO, USA 1:500) diluted in a solution containing 1% donkey serum and PBST and incubated for 48–72 h at 4 °C. After incubation, sections were washed in dsPBS 3 × 10 min and transferred to secondary Ab solution to be incubated overnight at 4 °C. Secondary Abs (Alexa 546 Donkey anti-Rabbit; Alexa 488 Donkey anti-goat, anti-mouse, or anti-sheep, all 1:400, Invitrogen, Oregon, USA) were diluted in a solution containing 1% donkey serum and PBST. Finally, sections were washed in PBS for 10 min and mounted using Vectashield mounting medium (Vector laboratories, Burlingame, CA).

### In situ hybridization combined with immunohistochemistry

Tissues were processed for combined fluorescent in situ hybridization and immunohistochemistry as previously described^7,10^, and based on^45^. The primer for antisense digoxigenin (DIG) riboprobe for vGluT2 was provided by the Jessell lab (Columbia University, USA), and the probe was kindly prepared by the Fawcett lab (Dalhousie University, NS, Canada). Briefly, sections were fixed in 4% PFA for 10 min at RT followed by washing in PBS 3 × 3 min. Sections were acetylated for 10 min in an acetic anhydride buffer and transferred to a hybridization solution (50% formamide, 5xSSC, 5xDenhardt’s, 250 µg/mL Baker’s yeast RNA, 500 µg/mL salmon sperm DNA; Sigma), initially without the probe at RT overnight. Sections were then heated in diluted hybridization solution with the probe for 5 min at 80 °C, then quickly transferred to ice before being incubated at 72 °C overnight in the solution. On day three, sections were incubated in 0.2× saline sodium citrate buffer (SSC) at 72 °C 2 × 30 min followed by equilibrating the sections in 0.2 × SSC for 5 min at RT. Sections were incubated in tris NaCl blocking buffer (0.1 M Tris chloride, 0.15 M NaCl, 0.5% blocking reagent; TNB) for 1 h at RT, then overnight in a solution containing donkey serum, sheep anti-DIG-POD (1:500 TNB; Roche), and rabbit anti-GFP antibody (1:500 TNB; Chemicon) at 4 °C. On day four, tyramide signal amplification (with AlexaFluor 555 or Cy3 (Perkin-Elmer), TSA kit #42, Invitrogen) was used to boost the fluorescent in situ signal. Sections were then incubated in anti-rabbit-Alexa 488 (Invitrogen) antibody for 3 h in PBS and mounted using Vectashield mounting medium (Vector laboratories, Burlingame, CA).

### Image acquisition

Confocal images were acquired with either a Zeiss LSM 710 Laser Scanning Confocal Microscope (Dalhousie) or Leica TCS SP5 (Columbia). For imaging synaptic puncta (VGlut1, VAChT), vibratome sections were imaged in Z stacks with a 20× or 63× objective (2 µm optical Z resolution). Images were captured either as 2D snapshots or 3D z-stacks with intervals of 0.8–1.88 μm and total Z-thickness up to approximately 30 μm. Pinhole size was set to 1 airy unit for all channels.

### Treadmill locomotion

These experiments were conducted at postnatal day 35–38 (15.9 ± 1.7 g) in control (cre negative with one or both floxed vGluT2 alleles, or cre positive with only one floxed vGluT2 allele) and experimental (Hb9-vGluT2^OFF^) mice. All mice were given tamoxifen as described above. Locomotor behaviour was studied on a treadmill (Cleversys, Inc.) equipped with a transparent belt and a high speed camera (Basler, USA). The treadmill belt and chamber were cleaned with Peroxigard prior to every experimental session. Mice were placed in a 161 cm^2^ chamber by an experimenter (LMK) not blinded to genotype, and gait was recorded with the supplied software (BCam Capture Version 2.00, Cleversys, Inc.) at a frame rate of 100 frames/s. Locomotor behaviour was examined in the afternoons of the light cycles of the animal at belt speeds of 15, 20, 27, and 30 cm/s. Faster speeds were attempted, however mice could not maintain locomotion at those speeds (e.g. 40 cm/s and 50 cm/s) for the full 20 s. The speeds were chosen to represent low, medium, and high speeds^46^. Locomotor activity was not facilitated through means such as electrical shocks or air puffs. Video recording started as soon as the treadmill speed was stable and lasted for 20 s per trial. Mice were allowed to rest for 1–2 min between each trial. In some cases, they were studied on a different day if they did not continuously locomote for 20 s at the test speeds. A total of 11 mice (6 female) were in the experimental group, and 12 mice (5 females) in the control group, which were littermates of the experimental mice. The sex of one mouse per group was not recorded. Power analysis revealed that a 50% increase of the step cycle CV (our primary measure) of 0.15 ± 0.05 in control mice with a = 0.05 and 80% power could be detected with 7 mice in the control and experimental groups.

### Gait analysis

Gait parameters were analyzed and recorded automatically using TreadScan Version 3.00 (Cleversys, Inc.). Prior to each experiment, a ruler was placed along the treadmill and imaged for scale calibration. An artificial foot model was created by drawing a polygon over the foot of interest at different frames, and saving the RGB ratios that represent each foot (Supplementary Fig. 1A). To exclude the instances when the mouse did not keep up with speed (e.g. when rearing or grooming), we included only sequences during which the mouse kept up with the treadmill for the full 20 s, and thus was near the front of the treadmill chamber. For each step, analysis parameters were obtained including: stance duration, which is the time during which the foot is in contact with the treadmill belt; swing duration, during which the foot is not in contact with the treadmill belt; cycle duration, which is the sum of stance and swing durations (Supplementary Fig. 1B); and stride length, which is defined as the distance travelled by the limb in a complete step cycle, and was calculated as (running speed * stride time) + displacement, where displacement could be positive or negative depending on the change of position of the foot in the camera frame. In addition, homologous coupling, defined as the coupling between the two hind limbs, homolateral coupling, defined as the coupling between ipsilateral limbs (e.g. right rear and right front), were calculated, with 50% meaning the limbs were out of phase. Rear track width, measured as the distance between the two hindlimbs measured perpendicular to the long axis of the body and is a measure of the hindlimb base of support was also calculated (Supplementary Fig. 1A,C). The reliability of each step was confirmed by examining all frames to ensure that each foot was adequately captured before exporting the raw data to an Excel file for analysis. The right hindlimb was used to quantify single limb parameters, both hindlimbs for homologous, and right fore- and hind-limbs for homolateral coupling (Supplementary Fig. 1).

### Statistics

For each step cycle parameter, normality was tested using the D'Agostino-Pearson test for the control vs experimental groups, and the Shapiro–Wilk test for the smaller sample sizes of females and males within these groups. The majority of distributions presented here passed these normality tests, so we proceeded with parametric tests to measure statistical significance. As the same animals were tested at different speeds, we used a repeated measures two way ANOVA (speed × genotype; sphericity assumed), but if there were missing values (i.e. if the animal failed to locomote at all speeds), a mixed effects model was used instead. For intra- and inter-group differences between females and males, we used unpaired one way ANOVA and for any significant differences, proceeded with Bonferroni multiple comparison tests with a single pooled variance to determine where the difference arose. For the comparison of swing or stance vs cycle duration (Fig. 7), linear regression analysis was done using ANCOVA-type calculations to compare^47^. All tests were done using Prism (GraphPad, San Diego, CA 9.1.1(225)). The experimental unit was taken as the mouse. P-values < 0.05 were considered to be significant. (Of note, because not all data sets were normally distributed, we also proceeded with a non-parametric Mann–Whitney U-test to compare control and experimental data, and a Kruskal–Wallis test for the 4 groups (genotype × sex), followed by a Bonferroni–Dunn test for any significant findings. Results were the same as the parametric analyses).

## Supplementary Information

Supplementary Information.

## References

[1] Grillner S, Jessell TM (2009). Measured motion: Searching for simplicity in spinal locomotor networks. Curr. Opin. Neurobiol..

[2] Alaynick WA, Jessell TM, Pfaff SL (2011). SnapShot: Spinal cord development. Cell.

[3] Kiehn O (2011). Development and functional organization of spinal locomotor circuits. Curr. Opin. Neurobiol..

[4] Arber S (1999). Requirement for the homeobox gene Hb9 in the consolidation of motor neuron identity. Neuron.

[5] Wichterle H, Lieberam I, Porter JA, Jessell TM (2002). Directed differentiation of embryonic stem cells into motor neurons. Cell.

[6] Hinckley CA, Hartley R, Wu L, Todd A, Ziskind-Conhaim L (2005). Locomotor-like rhythms in a genetically distinct cluster of interneurons in the mammalian spinal cord. J. Neurophysiol..

[7] Wilson JM (2005). Conditional rhythmicity of ventral spinal interneurons defined by expression of the Hb9 homeodomain protein. J. Neurosci..

[8] Kjaerulff O, Kiehn O (1996). Distribution of networks generating and coordinating locomotor activity in the neonatal rat spinal cord in vitro: A lesion study. J. Neurosci..

[9] Brownstone RM, Wilson JM (2008). Strategies for delineating spinal locomotor rhythm-generating networks and the possible role of Hb9 interneurones in rhythmogenesis. Brain Res. Rev..

[10] Bui T (2013). Circuits for grasping: Spinal dI3 interneurons mediate cutaneous control of motor behavior. Neuron.

[11] Caldeira V, Dougherty KJ, Borgius L, Kiehn O (2017). Spinal Hb9::Cre-derived excitatory interneurons contribute to rhythm generation in the mouse. Sci. Rep..

[12] Hnasko TS (2010). Vesicular glutamate transport promotes dopamine storage and glutamate corelease in vivo. Neuron.

[13] Rousso DL, Gaber ZB, Wellik D, Morrisey EE, Novitch BG (2008). Coordinated actions of the forkhead protein Foxp1 and Hox proteins in the columnar organization of spinal motor neurons. Neuron.

[14] Dasen JS, De Camilli A, Wang B, Tucker PW, Jessell TM (2008). Hox repertoires for motor neuron diversity and connectivity gated by a single accessory factor, FoxP1. Cell.

[15] Shneider NA, Brown MN, Smith CA, Pickel J, Alvarez FJ (2009). Gamma motor neurons express distinct genetic markers at birth and require muscle spindle-derived GDNF for postnatal survival. Neural Dev..

[16] Wilson JM, Cowan AI, Brownstone RM (2007). Heterogeneous electrotonic coupling and synchronization of rhythmic bursting activity in mouse Hb9 interneurons. J. Neurophysiol..

[17] Oliveira AL (2003). Cellular localization of three vesicular glutamate transporter mRNAs and proteins in rat spinal cord and dorsal root ganglia. Synapse.

[18] Tazerart S, Vinay L, Brocard F (2008). The persistent sodium current generates pacemaker activities in the central pattern generator for locomotion and regulates the locomotor rhythm. J. Neurosci..

[19] Orr BO (2020). Presynaptic homeostasis opposes disease progression in mouse models of ALS-like degeneration: Evidence for homeostatic neuroprotection. Neuron.

[20] Pun S (2002). An intrinsic distinction in neuromuscular junction assembly and maintenance in different skeletal muscles. Neuron.

[21] Yang X (2001). Patterning of muscle acetylcholine receptor gene expression in the absence of motor innervation. Neuron.

[22] Kramer ER (2006). Cooperation between GDNF/Ret and ephrinA/EphA4 signals for motor-axon pathway selection in the limb. Neuron.

[23] Li X-M (2008). Retrograde regulation of motoneuron differentiation by muscle β-catenin. Nat. Neurosci..

[24] Hess DM (2007). Localization of TrkC to Schwann cells and effects of neurotrophin-3 signaling at neuromuscular synapses. J. Comp. Neurol..

[25] Ju J (2016). Olig2 regulates Purkinje cell generation in the early developing mouse cerebellum. Sci. Rep..

[26] Rotolo T, Smallwood PM, Williams J, Nathans J (2008). Genetically-directed, cell type-specific sparse labeling for the analysis of neuronal morphology. PLoS ONE.

[27] Cheng TO (2010). William Dock, Willie Sutton and Sutton's law. Int. J. Cardiol..

[28] Anderson T (2012). Low-threshold calcium currents contribute to locomotor-like activity in neonatal mice. J. Neurophysiol..

[29] Kwan A, Dietz S, Webb W, Harris-Warrick R (2009). Activity of Hb9 interneurons during fictive locomotion in mouse spinal cord. J. Neurosci..

[30] Grillner S, El Manira A (2020). Current principles of motor control, with special reference to vertebrate locomotion. Physiol. Rev..

[31] Dougherty KJ, Ha NT (2019). The rhythm section: An update on spinal interneurons setting the beat for mammalian locomotion. Curr. Opin. Physiol..

[32] Eisen JS, Marder E (1984). A mechanism for production of phase shifts in a pattern generator. J. Neurophysiol..

[33] Rekling JC, Shao XM, Feldman JL (2000). Electrical coupling and excitatory synaptic transmission between rhythmogenic respiratory neurons in the preBotzinger complex. J. Neurosci..

[34] Ha NT, Dougherty KJ (2018). Spinal Shox2 interneuron interconnectivity related to function and development. Elife.

[35] Chang Q, Balice-Gordon RJ (2000). Gap junctional communication among developing and injured motor neurons. Brain Res. Brain Res. Rev..

[36] Ivanova A (2005). In vivo genetic ablation by Cre-mediated expression of diphtheria toxin fragment A. Genesis.

[37] Kriellaars DJ, Brownstone RM, Noga BR, Jordan LM (1994). Mechanical entrainment of fictive locomotion in the decerebrate cat. J. Neurophysiol..

[38] Juvin L, Simmers J, Morin D (2007). Locomotor rhythmogenesis in the isolated rat spinal cord: A phase-coupled set of symmetrical flexion extension oscillators. J. Physiol..

[39] Anderson TM, Ramirez JM (2017). Respiratory rhythm generation: triple oscillator hypothesis. F1000Res.

[40] Feldman JL, Kam K (2015). Facing the challenge of mammalian neural microcircuits: Taking a few breaths may help. J. Physiol..

[41] Ramirez JM, Baertsch N (2018). Defining the rhythmogenic elements of mammalian breathing. Physiology.

[42] Kristan WB, Katz P (2006). Form and function in systems neuroscience. Curr. Biol..

[43] Danner SM, Shevtsova NA, Frigon A, Rybak IA (2017). Computational modeling of spinal circuits controlling limb coordination and gaits in quadrupeds. Elife.

[44] Feil R, Wagner J, Metzger D, Chambon P (1997). Regulation of Cre recombinase activity by mutated estrogen receptor ligand-binding domains. Biochem. Biophys. Res. Commun..

[45] Vosshall LB, Amrein H, Morozov PS, Rzhetsky A, Axel R (1999). A spatial map of olfactory receptor expression in the Drosophila antenna. Cell.

[46] Beare JE (2009). Gait analysis in normal and spinal contused mice using the TreadScan system. J. Neurotrauma.

[47] Zar JH (1974). Biostatistical Analysis.

