# Supplementary material for: Elimination of glutamatergic transmission from Hb9 interneurons does not impact treadmill locomotion
Source: Sci Rep. 2021 Aug 6;11:16008. doi: 10.1038/s41598-021-95143-y (PMC8346588; doi:10.1038/s41598-021-95143-y)
Supplement: Supplementary file 1 — Supplementary Information. [file 41598_2021_95143_MOESM1_ESM.pdf]

Supplementary Information

Elimination of glutamatergic transmission from Hb9 interneurons does not impact treadmill locomotion

Lina M. Koronfel, Kevin C. Kanning, Angelita Alcos, Christopher E. Henderson, Robert M. Brownstone

Suppl Table 1: ARRIVE guidelines for behavioural experiments (Figures 5-7)

1. Study design	a. Experimental and control groups are littermates with and without genetic mutation. Males and Females secondarily analysed.<br>b. Experimental unit for behavioural studies is the mouse
2. Sample size	a. Sample size indicated for each analysis as well as the total number of mice<br>b. A 50% increase of step cycle CV from 0.15 ± 0.05 in control mice with $\alpha = 0.05$ and 80% power could be detected with 7 mice in the control and experimental groups
3. Inclusion and Exclusion	a. Mice that could not walk continuously for 20s on the treadmill at the given speed<br>b. See Methods for exclusion reasons<br>c. See Figures for the n's for each trial
4. Randomisation	a. Groups were based on genotype<br>b. Controls and mutants were housed together until weaning and then separated based on sex
5. Blinding	a. Experimenter was not blinded to genotype during treadmill locomotion experiments. Whole litters regardless of genotype were tested on any given day. Analysis was automated to prevent subjectivity
6. Outcome measures	a. Outcome measures are indicated in the Figures<br>b. The CV of step cycle duration was taken as the primary outcome
7. Statistical methods	a. Statistical methods as described in Methods<br>b. Normality tests as described in Methods
8. Experimental animals	a. Animal details, sexes, ages as in Methods<br>b. Details of genetic modification in Methods
9. Experimental procedures	a. Methods as described<br>b. Methods as described: all experiments done between 1100-1900, mice on a 12 hour light cycle beginning at 0700; some mice tested on a second day if they could not keep up for full 20s<br>c. Mice acclimatized to treadmill for several minutes before every trial, and rested for a few minutes between testing of different speeds<br>d. Rationale for procedures as discussed
10. Results	a. Descriptive statistics included in Figures and Table, all raw data points shown<br>b. All analyses reported; no effect sizes to report

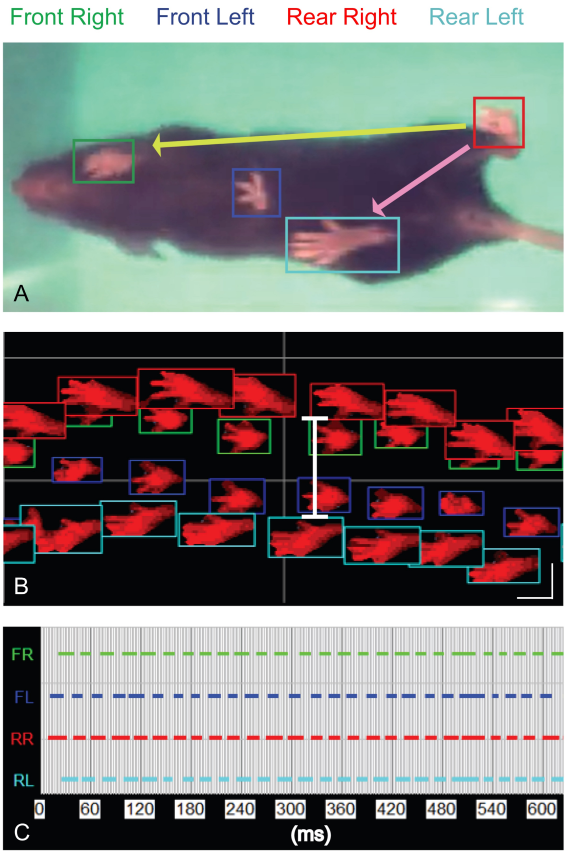

Supplementary Figure 1: Measuring locomotor parameters using TreadScan. A. Frame from video captured at 100 frames/s, cropped to show underside photograph of mouse on treadmill in chamber. Homologous coupling indicates coupling of the hind limbs (pink arrow), homolateral coupling indicates coupling between ipsilateral limbs (yellow arrow). B. Photogram of locomotor behaviour for a distance of 400 mm at treadmill belt speed of 27 cm/s. Rear track width is the horizontal distance between the hind limbs as measured perpendicular (bold white line) to the vertical axis of the body . Scale bar: 25 mm in x and y axes. C. Trace of cycle duration showing stance phases (coloured lines) for all 4 limbs for the first 600 ms of a test trial at 27 cm/s. Front Right (FR): Green, Front Left (FL): Blue, Rear Right (RR): Red, and Rear Left (RL): Cyan.
